# Development of a 3D‐printed single‐use separation chamber for use in mRNA‐based vaccine production with magnetic microparticles

**DOI:** 10.1002/elsc.202000120

**Published:** 2021-05-14

**Authors:** Lars Wommer, Patrick Meiers, Isabelle Kockler, Roland Ulber, Percy Kampeis

**Affiliations:** ^1^ Trier University of Applied Sciences Environmental Campus Birkenfeld Institute for biotechnical Process Design Hoppstädten‐Weiersbach Germany; ^2^ Technical University Kaiserslautern Institute of Bioprocess Engineering Kaiserslautern Germany

**Keywords:** 3D printing, high‐gradient magnetic separation, magnetic beads, mRNA‐based vaccines, single‐use application

## Abstract

Laboratory protocols using magnetic beads have gained importance in the purification of mRNA for vaccines. Here, the produced mRNA hybridizes specifically to oligo(dT)‐functionalized magnetic beads after cell lysis. The mRNA‐loaded magnetic beads can be selectively separated using a magnet. Subsequently, impurities are removed by washing steps and the mRNA is eluted. Magnetic separation is utilized in each step, using different buffers such as the lysis/binding buffer. To reduce the time required for purification of larger amounts of mRNA vaccine for clinical trials, high‐gradient magnetic separation (HGMS) is suitable. Thereby, magnetic beads are selectively retained in a flow‐through separation chamber. To meet the requirements of biopharmaceutical production, a disposable HGMS separation chamber with a certified material (United States Pharmacopeia Class VI) was developed which can be manufactured using 3D printing. Due to the special design, the filter matrix itself is not in contact with the product. The separation chamber was tested with suspensions of oligo(dT)‐functionalized Dynabeads MyOne loaded with synthetic mRNA. At a concentration of c_B_ = 1.6–2.1 g·L^–1^ in lysis/binding buffer, these 1 μm magnetic particles are retained to more than 99.39% at volumetric flows of up to 150 mL·min^–1^ with the developed SU‐HGMS separation chamber. When using the separation chamber with volumetric flow rates below 50 mL·min^–1^, the retained particle mass is even more than 99.99%.

**Abbreviations**
(m)RNA: synthetic polyadenylated ribonucleic acid
DLP: digital light processing
dT: deoxythymidine
HGMS: high‐gradient magnetic separation
HPLC: high performance liquid chromatography
L&E: leachables and extractables
mRNA: messenger ribonucleic acid
OD_600_
: optical density at λ = 600 nm
PBS: phosphate buffered saline
SDS: sodium dodecyl sulfate
SU: single‐use
TFF: tangential flow filtration
USP: United States Pharmacopeia

## 1 INTRODUCTION

### 1.1 mRNA vaccine manufacture

A new variant of vaccine production is becoming increasingly important with regard to viral infectious diseases. This involves the use of mRNA produced by cell culture methods. This mRNA is protected by special formulations and introduced into human cells, where it induces the expression of proteins and thus, triggers the immune response [1, 2, 3, 4, 5]. In the search for new vaccines, laboratory protocols that use functionalized magnetic beads for the purification of mRNA out of cell lysates are often used. For this purpose, magnetic particles with deoxythymidine functionalization (oligo(dT)), with a sequence of 14–25 thymine bases are utilized [6, 7]. Hybridization of the mRNA to the oligo(dT) magnetic particles takes place specifically, due to the base adenine complementary to thymine [6, 7]. Only mRNA molecules have an adenine chain with 40–250 units at the 3′ end. This does not exist on RNA or DNA molecules, so selective sorption occurs by hybridization of mRNA on the oligo(dT) magnetic particles. After separation of the particles loaded with mRNA in the magnetic field and removal of impurities with several washing steps, elution of the mRNA can be initiated by increasing the temperature. Here, successive multiple magnetic separations and resuspensions are involved, for which suitable millilitre‐scale laboratory protocols have been developed, some of which are automated [6, 7, 8].

**PRACTICAL APPLICATION**

In the event of a virus‐triggered pandemic, mRNA vaccines appear promising for rapid containment. Protocols for mRNA isolation at laboratory scale are often based on magnetic beads. Here, after synthesis in cell culture, mRNA is selectively hybridized with oligo(dT)‐functionalized magnetic beads. Isolation then proceeds through several washing steps and the final elution step, each with magnetic separation. Up‐scaling is required to produce vaccine quantities for clinical trials. High‐gradient magnetic separation (HGMS), in which magnetic beads are separated in a flow‐through process in a separation chamber with magnetizable matrix, is suitable for this purpose. For use in mRNA production, an HGMS separation chamber was developed that can be 3D‐printed from a USP Class VI certified material. Due to the design, only this material is in contact with the product. Excellent separation performance was achieved, even when separating a particle system with a very small mean particle diameter.

One problem is the scale‐up of mRNA purification from laboratory scale to production scale, which is necessary to produce the amount of vaccine needed for clinical trials. Clinical trials are divided into phases I to IV depending on the number of patients involved. Already in phase III, each vaccine candidate must be available for 1000–2000 participants [9]. Currently, mRNA is purified in large‐scale production by preparative HPLC, preferably as ion pair reversed‐phase chromatography [10, 11, 12, 13, 14, 15]. This is performed with a porous stationary phase and polar solvents such as acetonitrile and/or methanol in water as the mobile phase. Alternatively, chromatography with cellulose as the stationary phase can also be considered [10]. New approaches are performed with anion exchange and hydrogen bonding chromatography [12]. Regardless of the type of chromatography, however, the liquid to be processed must always be free of particles due to the characteristics of the system. This requires an additional solid/liquid separation step such as tangential flow filtration (TFF). Purification of mRNA by TFF and HPLC results in product losses in the range of 20–50%. [16, 17].

### 1.2 High‐gradient magnetic separation

If it was possible to use oligo(dT) magnetic particle‐based purification of mRNA for clinical trials, there would be no need for a technology change in scale‐up from laboratory scale to process scale. With high‐gradient magnetic separation (HGMS), the required process technology is available. It has already been used by various working groups in the field of biotechnology [18, 19, 20, 21, 22, 23, 24, 25, 26, 27, 28, 29, 30, 31]. The magnetic separators used at HGMS, which operate in the flow‐through mode (“magnetic filters”), were and are developed in particular by Franzreb [20, 21, 22] and in own work [25, 26, 30]. The advantage of this technique in the production of mRNA arises from the fact that the laboratory protocols used by several users in research can be transferred 1:1 to production. This results in time savings as there is no need to develop standard operating procedures for solid‐liquid separation and chromatography. This could shorten the time to start phase II and III clinical trials. In the event of a pandemic, this is considered to be far more important than the possible economic disadvantage of a magnetic particle‐based process. In addition, HGMS enables single‐stage solid/solid/liquid separation of the multicomponent suspension. This means that a solid/liquid separation step is not necessary here. Since mRNA degrades relatively fast [14], every time saving in the process flow would be beneficial for product quality.

In high‐gradient magnetic separation, the magnetic field distribution and thus, the gradient of the magnetic field is significantly influenced by the material of the filter matrix as well as its structure, arrangement, and size [32, 33, 34, 35]. Since the matrix normally comes into contact with the suspension, it should have chemical resistance and corrosion resistance, as well as meeting leachables and extractables (L&E) criteria in biopharmaceutical applications. Therefore, the material is usually limited mainly to magnetizable stainless steels. In the widely used structured filter matrices, axial as well as transverse rod arrangements, and linear or rhombic designs are possible [32]. Rhombic arrangements were characterized by higher loading capacities for both axial and transverse placement. Simulations using *COMSOL Multiphysics* software revealed the best separation performance for transverse rhombic matrices [35].

### 1.3 Single‐use technology

Single‐use (SU) applications are increasingly used for the production of biopharmaceuticals to avoid cross‐contamination between batches of active ingredients and to enable more flexible production [36]. Disposable chromatography columns, normal flow, and tangential flow filtration units are yet commercially available [37, 38, 39, 40]. With a suitable separation chamber, the HGMS technology can also be carried out in the form of a single‐use application. Shaikh et al. [30] already tested disposable plastic bags equipped with steel wool for HGMS. Thanks to the single‐use approach, the time‐consuming cleaning in place (CIP) and sterilization in place (SIP) between different batches to avoid cross‐contamination are eliminated in the SU‐HGMS.

### 1.4 3D printing

The 3D printing process was chosen for the manufacture of a SU‐HGMS separation chamber. This allows geometries of one‐piece components with undercuts to be realized that are not possible with conventional manufacturing processes. In addition, the flexibility of 3D printing makes it easy to optimize geometries. The use of 3D‐printed parts in downstream processing hasn't been explored extensively [40]. 3D‐printed stationary phases with ordered morphology are tested for chromatography [41, 42]. Recently, research is extended using 3D printing for membrane separation, desalination, and water purification applications [43, 44]. Kolczyk‐Siedlecka et al. [45] developed a 3D‐printed microflow device for the magnetic enrichment of rare‐earth metal ions. Frodsham [46] used an HGMS separation chamber for hemofiltration of malaria infected red blood cells. To the best of our knowledge, the use of 3D printing in production processes has not been reported in the literature.

In this work, a 3D‐printed HGMS separation chamber was developed for SU applications. It was designed for use in the production of mRNA‐based vaccines and is presented here as well as its evaluated separation performance. For the development and testing of the new SU‐HGMS separation chamber, a particle system was deliberately chosen in the form of Dynabeads MyOne. Due to its very small mean particle diameter of 1 μm, it places very high demands on the separation performance of the magnetic separation (see Section 3.3). Therefore, (m)RNA‐loaded oligo(dT)‐functionalized magnetic beads prepared according to Section 2.2.2 were used in the experiments. In addition, the buffer system in which the magnetic particles are suspended, also plays a role (see Section 3.1 and Section 3.3.3). Therefore, the so‐called lysis/binding buffer and the PBS buffer, which are important in mRNA‐based vaccine production (see Section 2.2.3), were used and compared with the reference system, deionized water. In the magnetic separations performed, the volume flow rate was varied, as this is an important process parameter of the overall process.

## 2 MATERIALS AND METHODS

### 2.1 3D printing using Digital Light Processing

The separation chamber was 3D‐printed using the *Vida* 3D printer (EnvisionTEC GmbH) with a layer height of 50 μm at a temperature of 23°C. The maximum possible component dimensions are 139 × 78 × 100 mm (W x D x H). In the 3D printing process via Digital Light Processing (DLP), the UV light emitted by a projector causes the polymerization reaction of a photoreactive resin. The pixel width of the projector with a power of 330 W was 73 × 73 μm at a resolution of 1920 × 1080.

#### 2.1.1 Software‐based design

The design of the separation chamber was carried out by means of Computer Aided Design (CAD) with the software *Siemens NX 1859*. When exporting the *.stl‐files, a lateral tolerance of 0.08 and an angle tolerance of 1° were selected. The component generated in this way was sliced by *Perfactory Rapid Prototyping 3.2.3377.1712* software into individual layers equivalent to the selected layer height. The layers were transferred to the printer as image files and printed one after the other [47]. Depending on the geometry and nominal dimension of the component to be printed, a suitable alignment must be made for the printing process. Support structures may be required to establish contact between component regions and the building platform or to increase their contact areas (see Section 3.2). These were generated using the software *Materialise Magics 20.2*. The software *Comsol Multiphysics 4.3.0.151* was used for Computational Fluid Dynamics (CFD) as described in [25]. Due to the symmetry of the separation chamber, the CFD calculation was performed with a halved geometry. A predefined mesh with “finer” meshing optimized for fluid dynamics was chosen. “Laminar flow” with “compressible flow Ma <0.3″ was selected. The density of the solution was set to 1005 kg·m^–3^ and its dynamic viscosity to 1.4·mPa·s. Outflow condition was set to “pressure, no viscous stress” with a pressure of 10^5^ Pa. Wall boundary condition was set to “no‐slip,” resulting in flow velocities of 0 m·s^–1^ at the walls of the separation chamber.

#### 2.1.2 USP Class VI certified 3D printing material

With E‐Shell 600 from DeltaMed GmbH, a material certified according to USP Class VI and thus, biocompatible according to ISO 10993, was chosen for 3D printing of the separation chamber. It is resistant to common sterilization procedures and chemicals. E‐Shell 600 is a photoreactive resin containing 60–80% acrylic monomer, 10–25% tetrahydrofurfuryl methacrylate, 5–20% urethane dimethacrylate and <1% diphenyl(2,4,6‐trimethylbenzoyl)phosphine oxide [48, 49].

### 2.2 Magnetic separation

#### 2.2.1 High‐gradient magnetic separation

**FIGURE 1:**
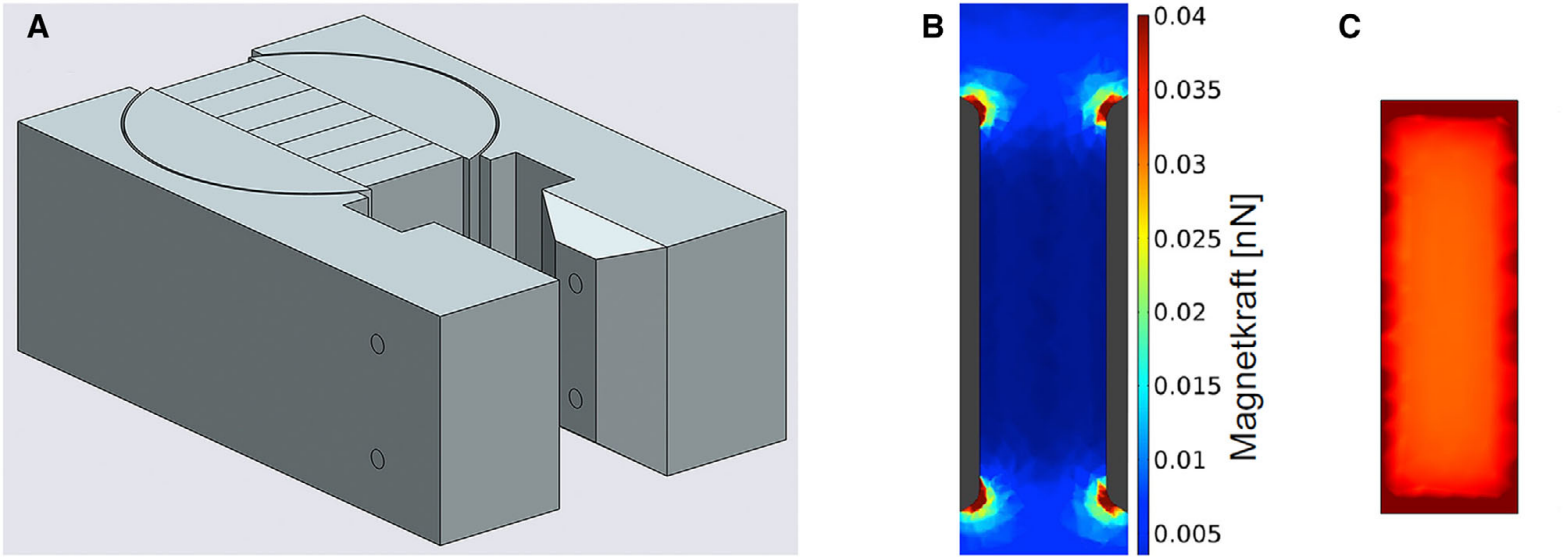
Modified yoke (A) and magnetic field of the Steinert HGF‐10 magnetic separator, side view (B), front view (C)

To carry out the magnetic separation, an existing plant was used [26, 50]. The core of the plant is a magnetic separator HGF‐10 (Steinert Elektromagnetebau GmbH). The magnetic field generated by the magnetic separator using a modified yoke is shown in Figure 1 [50]. An Ismatec MCP peristaltic pump (IDEX Health & Science GmbH) was used with a Masterflex Easy‐Load 2 dual‐channel pump head (Cole‐Parmer GmbH) to deliver the magnetic particle suspensions. The two parallel pump tubes Masterflex C‐Flex (Cole‐Parmer GmbH) had an inner diameter of 6 mm. All other hoses were made of polyurethane (Riegler & Co. KG) with an inner diameter of 4 and 6 mm. After insertion of the SU‐HGMS separation chamber into the yoke of the magnet, connections between separation chamber and tubing system were made using Tri‐Clamp. Then, the magnetic field was switched on and the system was filled with deionized water or the appropriate buffer until it was free of air bubbles.

The magnetic particle suspension was in each case in a 1 L or 3 L measuring beaker made of styrene‐acrylonitrile copolymer (VITLAB GmbH), which served as feed vessel. Homogenization was carried out in the beaker using a four‐bladed propeller stirrer with a diameter of d = 5 cm (R1342, IKA‐Werke GmbH & Co. KG) at a suspension volume V < 1 L or with a diameter of d = 10 cm (R1345, IKA‐Werke GmbH & Co. KG) at a suspension volume of V >1 L. With the stirrer drive RW 16 basic (IKA‐Werke GmbH & Co. KG) 500 rpm and 220 rpm were applied, respectively.

Ultrasonic treatment was performed before each magnetic separation (except for the experiments in Section 3.3.2) to ensure that the initial particle size distribution was present. For this purpose, magnetic particle suspensions of 400 mL were stirred for 10 min. Then, ultrasonic treatment was performed according to Section 2.2.4. The desired magnetic particle concentration was adjusted by adding another liquid. From the feed vessel, the suspensions were pumped through the separation chamber at the selected volume flow rates. The filtrate of the HGMS was collected in a beaker.

Cleaning of the separation chamber between experiments was first performed by backflushing about 300 mL of the filtrate with 600 mL·min^–1^ with the magnetic field turned off. Then, the filtrate was pumped at 700 mL·min^–1^ in the filtration direction with a two‐phase flow of liquid and air as described by König et al. [28]. Any remaining particle residues were removed with new liquid in the direction of filtration.

#### 2.2.2 Particle system

Dynabeads MyOne Carboxylic Acid (Life Technologies AS) was selected as particle system. These are uniformly spherical and monodisperse magnetic particles with a diameter of 1.05 μm ± 0.03 μm [51] and a functionalization of 0.6 mmol carboxyl groups per gram of particles [52]. Their physical and chemical properties are described in [53, 54, 55, 56, 57]. The Dynabeads MyOne Carboxylic Acid were coupled with oligonucleotides with a sequence of 25 thymine bases according to the protocol [57], since these 1 μm particles are not commercially available in oligo(dT)‐functionalized form. For this purpose, amino‐functionalized oligonucleotides were purchased from Invitrogen Life Technologies AS. Between the amino group at the 5′ end, 6 carbon atoms were attached as spacers, followed by 25 bases of thymine in the 3′ direction. The molecular weight was 7724 g·mol^–1^.

The oligo(dT)_25_‐functionalized magnetic particles prepared in this way were then loaded with mRNA. In order to have a sufficient amount of mRNA available, synthetic polyadenylated RNA—so to speak as “synthetic mRNA” referred to as (m)RNA in the following was prepared as follows: *E. coli BW3110* with plasmid pJOE 4056.2_6His_eGFP [58] from 300 μL glycerol cryoculture was prepared in an overnight culture in 150 mL LB medium containing 150 μL 10% (w/v) ampicillin in a shake flask and incubated at 37°C and 120 rpm for approximately 16 h. All subsequent steps took place in accordance with the respective instructions of the kits mentioned below. The *GeneJET Plasmid Miniprep* kit (ThermoFisher Scientific Inc.) was used for plasmid DNA isolation. Cutting of the plasmid was performed using the HindIII‐HF restriction enzyme (New England Biolabs GmbH). The DNA was then in linearized form and purified using the *DNA Clean & Concentrator‐5 (Capped)* kit (Zymo Research Europe GmbH). Through the *AmpliScribe T7 Flash Transcription* kit (Lucigen Corporation), the DNA was translated into complementary RNA of approximately 900 bases in length. Purification of RNA was performed via precipitation with 3.854 g ammonium acetate (Carl Roth GmbH & Co. KG, ≥97%) in 10 mL ultrapure water. The purified RNA was then polyadenylated using the *A‐Plus Poly(A) Polymerase Tailing* kit (Cellscript LLC). Hybridization of the polyadenylated RNA was performed according to the prescription of the *mRNA Purification* kit (ThermoFisher Scientific Inc.) for commercially available 2.8 μm oligo(dT)_25_ Dynabeads. Hybridization was detected based on elution of a sample of a larger hybridization mixture using the UV/Vis spectrophotometer (DS‐11, DeNovix Inc.).

#### 2.2.3 Buffers for the preparation of the magnetic particle suspensions

The lysis/binding buffer consisted of 15.76 g Tris‐HCl (AppliChem GmbH, ≥99%), 21.197 g LiCl (Carl Roth GmbH & Co. KG, pure ≥98.5%) and 10 g SDS (Carl Roth GmbH & Co. KG, ultrapure) in 1 L deionized water. pH of 7.5 was adjusted with 1 M NaOH (Carl Roth GmbH & Co. KG, 1 N measured solution). Note: The substances DTT and EDTA, which are often used in lysis buffers, were omitted because their use in interaction with Dynabeads is not recommended [59].

PBS buffer was composed of 0.294 g NaH_2_PO_4_ * 2H_2_O (Merck KGaA, ≥99%), 1.44 g Na_2_HPO_4_ * 2 H_2_O (Carl Roth GmbH & Co. KG, ≥98%) and 8.78 g NaCl (AnalaR Normapur, >99.5%) in 1 L deionized water. Note: Commercially available oligo(dT)‐functionalized 2.8 μm Dynabeads for mRNA purification are supplied in this buffer [60].

#### 2.2.4 De‐agglomeration of magnetic particle suspensions

An ultrasonic sonotrode (HD 2200, GM 2200, KE 76, Bandelin electronic GmbH & Co. KG) was used for de‐agglomeration of magnetic particles. The operating frequency was 20 kHz at a power of 200 W. The sonotrode tip was immersed 3 cm into the 0.4 L magnetic particle suspension, which was sonicated for 1 min at an amplitude of 19–23%, corresponding to a power input of 52.3 kW·m^–3^. After the ultrasound treatment was performed, the respective magnetic particle suspension was replenished to the required volume to achieve a magnetic particle concentration of c_B_ = 1.6–2.1 g·L^–1^.

### 2.3 Analytical methods

#### 2.3.1 Measurement of particle concentration by means of turbidity measurement

In the case of low‐concentration particle suspensions, predominantly in the filtrate, the concentration determination via dry mass measurement reaches its detection limit. Therefore, to determine the concentration of particle suspensions, turbidity measurements were performed using an UV/Vis spectrometer (Genesis 10, Thermo Scientific Inc.) at a wavelength of λ = 600 nm. The measurements took place in 10 × 4 × 45 mm half‐micro cuvettes made of polystyrene (Sarstedt AG & Co. KG). If necessary, samples were diluted with deionized water or respective buffer. The filtrate produced by the HGMS was measured undiluted.

#### 2.3.2 Determination of the particle size distribution

A laser particle sizer with associated small‐volume liquid dispersion unit (Analysette 22 MicroTec, Fritsch GmbH) was used for particle size analysis. The device has a green laser with a wavelength of λ = 532 nm for particles in the fine range and an IR laser with λ = 850 nm for particles in the coarse range. To fully capture the particle size distribution of a sample, both lasers were used, allowing a range of 101 size classes between 0.09 μm and 2 mm to be imaged. A background measurement was performed before each measurement. The evaluation was performed using the program *MaS control V1.00.009* according to the Fraunhofer theory, since the optical properties of the Dynabeads (refractive index and absorption coefficient) were not known. “Very narrow” was chosen as the calculation model because it has the best ratio of smoothing to root‐mean‐square error for the particles used. The amount of sample used varied depending on the concentration, since a beam absorption between 10% and 15% was aimed for. From the cumulative distribution, the median values of the volume distribution d_3,50_ were determined in the software.

#### 2.3.3 Contact angle measurements

Contact angle measurements were performed by adding 5 μL fluid as a droplet onto the surface of the part to be examined. A video with a frame rate of 60 fps was taken with a camera (i‐Speed 220, iX Cameras Ltd.) and the associated software *Control 2 Series 1.0.9*. The contact angle was determined from the images in triplicate using imaging software *ImageJ 1.52n*.

## 3 RESULTS AND DISCUSSION

### 3.1 Constructive design of the filter chamber

The SU‐HGMS separation chamber was designed in such a way that additive manufacturing with E‐Shell 600 is possible. The special requirements of additive manufacturing using the DLP process were taken into account in the design of the filter chamber, and the filter chamber was designed with regard to high particle retention with the most complete magnetic particle recycling possible.

The separation chamber was adapted to an existing HGMS system designed for feed volumes >400 mL which is described in [26, 50]. The external dimensions of the filter chamber of 44 × 50 × 128 mm have been retained. The filter matrix was extended to the entire length of the separation chamber, in contrast to Shaikh [50] who used a matrix length of 80 mm. The rectangular flow cross‐section has the dimensions of 26 × 18 mm over the entire length without internals. The liquid inlet and liquid outlet establish the connection with the tubing system via Tri‐Clamp on the one hand and enabled the transition from a circular area to a rectangular cross‐section on the other hand.

A rhombic bar arrangement was selected as filter matrix geometry, in which the distance from bar center to bar center was 3.7 mm. This resulted in 19 rod rows each with five or six rods. Rod sleeves were printed as continuous hollow cylinders of a diameter of 1.7 mm with a wall thickness of 0.5 mm. The magnetizable material for forming the magnetic field gradients can be inserted into these hollow cylinders as rods. Due to this special design solution, only the USP Class VI certified material comes into contact with the product. Because of the wetting properties and low surface roughness of the polymer, particle recovery can be improved compared to machined stainless steels (see Section 3.3.4). The filter matrix was made of stainless steel 1.4016 (X6Cr17) and polished after manufacture. After wetting with water (see Section 2.2.2), the contact angle was 85 °. The separation chamber made of E‐Shell 600 has two different surface properties due to the 3D printing direction relative to the orientation of the part on the build platform. Therefore, two different contact angles of 47 ° and 68 ° were obtained by wetting the resin surfaces with water. Thus, E‐Shell 600 shows increased hydrophilicity compared to machined stainless steel. Wetting with PBS buffer resulted in contact angles of 32 ° and 66 °. When lysis/binding buffer was used, a further reduction to 30 ° and 40 ° was observed. These results show that the use of buffers instead of deionized water leads to better wettability of the resin.

**FIGURE 2:**
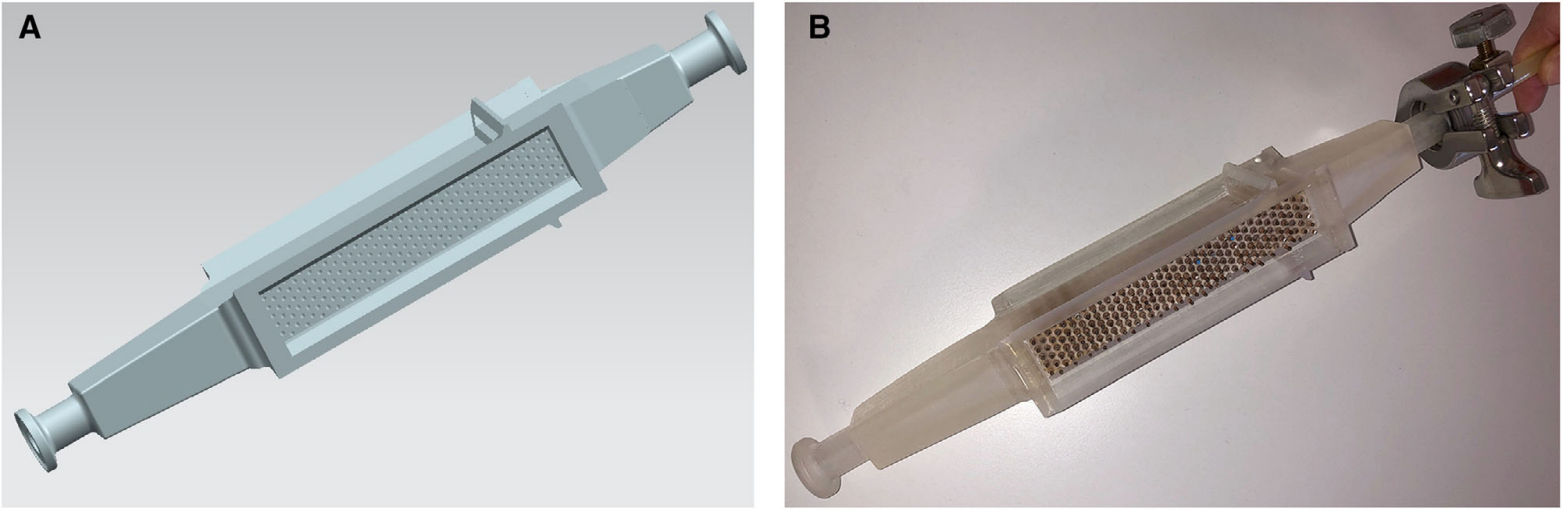
CAD model of the separation chamber including liquid inlet and liquid outlet with Tri‐Clamp connectors (A), 3D‐printed SU‐HGMS separation chamber equipped with metal rods (B)

In addition, the simple technique of inserting the metal rods into the rod sleeves makes it possible to automate the insertion of the filter matrix. With an appropriate rod holder, all rods can be inserted together with a pick‐and‐place robot. In the same way, the rod holder including all rods can be removed automatically after use, making it available again for the next application. This means that only the plastic housing of the separation chamber would have to be replaced for SU applications. Figure 2 shows the CAD model and the 3D‐printed, fully assembled separation chamber fitted with a total of 5434 mm of magnetizable rods made of steel (material number 1.5125, diameter 1.6 mm).

**FIGURE 3:**
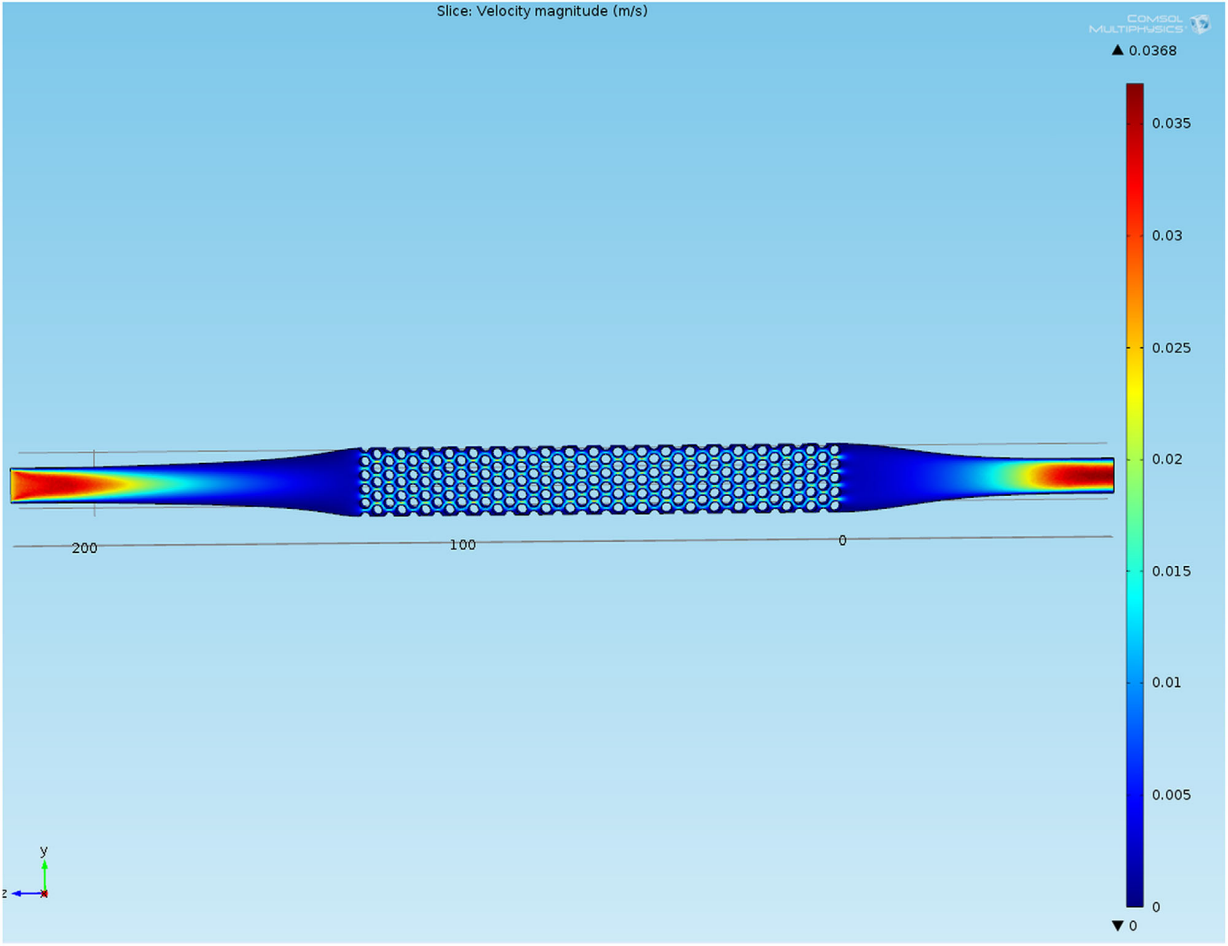
CFD simulation of the separation chamber at a volumetric flow rate of 100 mL·min^–1^ (flow direction from left to right, flow velocities given in m·s^–1^)

During development, CFD calculations were also performed as described in Section 2.1.1. Figure 3 shows the CFD simulation of the flow profile in the center of the separation chamber according to a flow rate of 100 mL·min^–1^ (corresponding to a flow velocity of 0.024 m·s^–1^ at the liquid inlet). As can be seen, the flow velocity is very uniform over the entire flow cross‐section in the separation chamber due to the selected geometry.

Due to the limited size of the building platform of the 3D printer used, the liquid inlet and liquid outlet had to be fabricated separately and glued to the separation chamber as described in Section 3.2. Using a 3D printer with a larger building platform, the entire separation chamber could be fabricated in one step, completely eliminating the need for subsequent gluing.

### 3.2 3D printing with USP Class VI certified material

**FIGURE 4:**
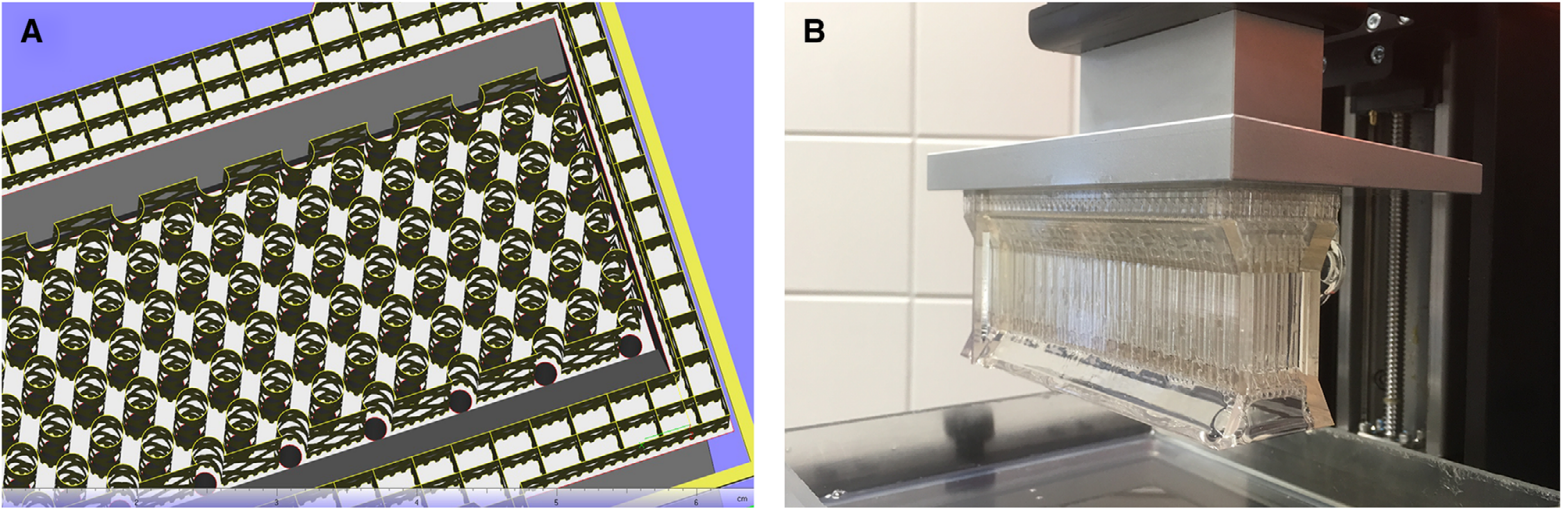
Use of block and contour supports for 3D printing of the separation chamber in Materialise Magics 20.2 software (A), separation chamber with supports directly after 3D print (B)

All product‐contacting materials within biopharmaceutical production must meet specific limits regarding leachables and extractables (L&E). These are defined in ISO 10993. The materials used should be resistant to sterilization processes, solvents, and other chemicals. Since E‐Shell 600 is a suitable material for 3D printing using the DLP process and has a United States Pharmacopeia certificate (USP Class VI), this 3D printing technique was selected. The entire length of the filter chamber was printed in a single job. The liquid inlet and liquid outlet were fabricated separately in a second job. In 3D printing using the DLP process with E‐Shell 600, only overhangs in the range of about 1 mm can be printed from layer to layer, since the 3D printing material is not yet fully cured after the exposure step in the printing process. For overhangs >1 mm, the adhesion force between the new layer and the fluid reservoir may exceed the adhesion force between the new layer and the component, resulting in its detachment and thus, defective components in print. To avoid this, support structures are necessary. After removal of the support structures, cured residues inevitably remain, which must be avoided inside the separation chamber to prevent magnetic particle build‐up that cannot be removed and would make recycling difficult. On the outside of the separation chamber, cured material residues are not critical. Square block supports were used on continuous surfaces (see Figure 4). No support structure has been used for the sloped areas because the path difference of the individual layers is <1 mm. For the structured filter matrix, the support structure was adapted to the arrangement of the rod shells by using contour supports.

Immediately after completion of the 3D printing, post‐treatment began. The components were rinsed with isopropyl alcohol (VWR, AnalaR Normapur, 99.9%) and manually removed from the building platform. Support structures were also removed, after which the components were treated in isopropyl alcohol for 3 min in an ultrasonic unit (P‐DW 20 US, Schmitt Ultraschalltechnik GmbH). They were then dried for 30 min at a temperature of 37°C in an incubator (BD 56, Binder GmbH). This was followed by post‐exposure of the components to UV light to achieve mechanical stability of the material. For this purpose, the components were placed in a post‐exposure unit equipped with two LED emitters for 1 min. The light sources had three sets of chips with wavelengths of λ_1_ = 395 nm, λ_2_ = 410 nm, and λ_3_ = 450 nm at a power of 30 W each.

As mentioned above, the liquid inlet and liquid outlet had to be fabricated separately and glued to the separation chamber. This was also done with E‐Shell 600, which means that the complete component, including the adhesive, is made of a single material. Therefore, the contact surfaces were thinly coated with E‐Shell 600, joined together and placed in the post‐exposure unit. When exposed for more than 0.5 min, the material hardens at the gluing surface and forms a liquid‐tight bond.

The separation chamber presented here is intended to shorten the lead time for clinical studies by transferring the laboratory protocols 1:1 to the production quantity required for this purpose. The number of separation chambers required for this purpose can still be described as “small‐batch production.” Therefore, the production output of the available DLP 3D printers is still completely sufficient for this application. A major advantage of 3D printing is that the geometry can be changed quickly if separation problems should arise in the field, e.g. due to a changed magnetic particle system. Then, the filter chamber can be quickly revised without the need for new manufacturing tools (e.g. injection molds). Furthermore, on‐demand production can be realized. Due to the (still) high costs for the required magnetic particles, the filter chamber presented here is not aimed at large‐scale production. Should the magnetic particle‐based mRNA manufacturing process actually be used there in the future, alternative manufacturing processes (e.g. injection molding) can still be considered.

### 3.3 Separation experiments

The SU‐HGMS filter chamber separation tests were performed using the HGMS system described in Section 2.2.1. Suspensions consisting of the (m)RNA‐loaded oligo(dT)_25_‐functionalized Dynabeads (see Section 2.2.2) and the buffers or deionized water as reference (see Section 2.2.3) were used. The balance of forces on the magnetic particles results in small particles being more difficult to separate in an HGMS due to the higher frictional force (surface force) compared to the magnetic force (volume force). Therefore, a continuous loss of fine particle fractions may occur even before the capacity of the separation chamber has been reached [61]. Agglomerates, on the other hand, are easier to separate. When a magnetic field is applied, the magnetic particles form agglomerates [53, 56, 62, 63, 64, 65]. Particle‐particle interactions support this agglomeration. As a result, shorter separation times can be achieved [53], which can favour better separation performance [62, 64, 65]. The surface loading of the particles could contribute to the agglomeration behavior. It could be increased by attracting forces of complementary bases of mRNA molecules between different particles [66, 67, 68, 69]. Repulsion forces can be expected when the particles are solubilized by detergents like SDS.

#### 3.3.1 Determination of concentration in the magnetic filtrate by means of turbidity measurement

**FIGURE 5:**
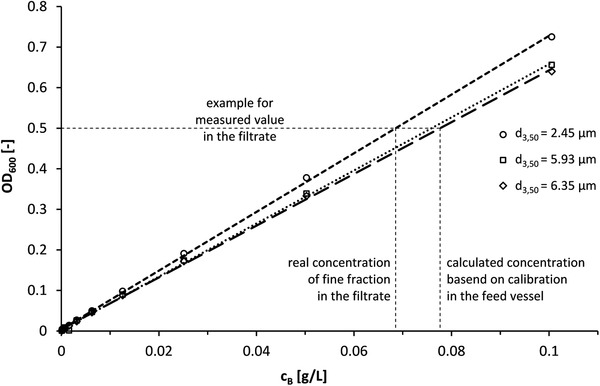
Decrease in turbidity (OD_600_) of a suspension of (m)RNA‐loaded oligo(dT)_25_ Dynabeads MyOne (c_B_ = 2.0 g⋅L^–1^) in the feed vessel caused by an increase in median particle size

When using turbidity measurement to determine the magnetic particle concentration, it is assumed that the particle size distribution does not change. However, this is not the case as mentioned above. Thus, the actual magnetic particle concentration present can only be approximated by this method. It is helpful, however, that the calibration lines for magnetic particle suspensions with smaller particle diameters have higher slopes than those of suspensions with agglomerated magnetic particles (see Figure 5).

Here, the median particle diameter increased from d_3,50_ = 2.452 μm over d_3,50_ = 5.929 μm to d_3,50_ = 6.353 μm while stirring in the feed vessel at 500 rpm. The slope of the corresponding calibration line of the turbidity decreased from 7.236 to 6.546 to 6.380 L·g^–1^. Usually, turbidity calibrations were made with the suspension in the feed vessel and its particle size distribution. When carrying out HGMS separations, small particles in particular cannot be retained by the magnetic filter due to the force balance on the particles as mentioned above. This means that the particle sizes in the magnetic filtrate are generally smaller than in the feed vessel. With smaller particle size in the filtrate compared to the suspension in the feed vessel, a larger particle loss in the filtrate of the HGMS separation is calculated than actually occurred (see Figure 5). Therefore, this measurement method is nevertheless used in the development and optimization of magnetic separators [50].

#### 3.3.2 Separation tests without ultrasonic de‐agglomeration in the feed vessel

To characterize the HGMS filter chamber, separation experiments of the (m)RNA‐loaded oligo(dT)_25_ Dynabeads (c_B_ = 1.6–2.1 g·L^–1^) were performed in deionized water with different volume flows. Here, the suspensions were dispersed only by means of the stirring system in the feed vessel. A suspension of 0.8 L was stirred for 1 h before the experiment. Magnetic separation was then performed as described in Section 2.2.1. The median particle diameter in the feed vessel increased from experiment to experiment from d_3,50_ = 2.035 μm over d_3,50_ = 4.792 ± 0.082 μm to d_3,50_ = 5.386 ± 0.397 μm. Thus, particle agglomeration had occurred.

**FIGURE 6:**
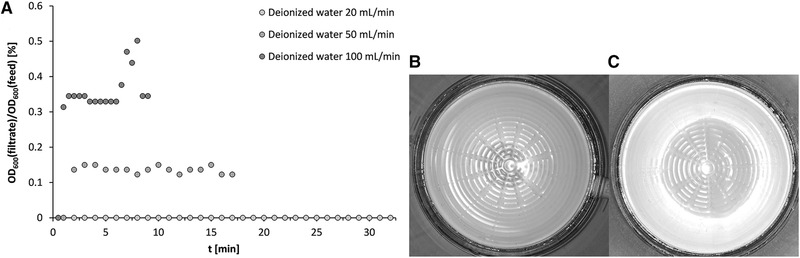
Course of filtrate turbidity normalized to feed suspension during HGMS filtration of non‐ultrasonic‐treated (m)RNA‐loaded oligo(dT)_25_ Dynabeads MyOne (c_B_ = 1.8 g·L^–1^) in deionized water as a function of volume flow rate (A), residues of sterile‐filtered magnetic filtrate on 0.2 μm filter after HGMS with 20 mL·min^–1^ (B) and 50 mL·min^–1^ (C)

Figure 6 shows the course of the turbidity in the magnetic filtrate. No particles could be detected in the filtrate via the measurement of turbidity at a flow rate of 20 mL·min^–1^ (see Figure 6A). The concentration was thus below the detection limit of 0.3 mg·L^–1^. In addition, the complete filtrate was filtered through a 0.2 μm BT 25 bottle‐top filter (Sarstedt Inc.). Afterwards, no brownish particle residue was visually detectable (see Figure 6B). With a volumetric flow rate of 50 mL·min^–1^, some particles entered the filtrate, which could be detected via the turbidity measurement. Nevertheless, no particle residues were visible to the naked eye after the filtrate was analogously filtered through a 0.2 μm bottle top filter (see Figure 6C). Therefore, only extremely low particle loss occurs at both volume flows. A volume flow rate increased to 100 mL·min^–1^ caused the immediately recognizable turbidity of the filtrate and thus, to a higher particle loss. The developed separation chamber is nevertheless well suited for the separation of magnetic particle suspensions with partially agglomerated fractions. At volumetric flows of up to 100 mL·min^–1^, the magnetic particles are retained to more than 99.68%.

#### 3.3.3 Influence of buffer chemicals after ultrasonic treatment on the separation efficiency

Purification of mRNA from cell lysate involves several washing steps and buffer changes. Therefore, the suitability of the separation chamber in selected buffer systems was investigated in the following. The main focus was on the so‐called lysis/binding buffer and the PBS buffer. The former is used for the release of mRNA from cells and the subsequent binding of mRNA to magnetic particles. The latter is used in the storage of Dynabeads. Deionized water served as the reference system, since magnetic separators are often designed with it [50]. Due to the buffer components, the zeta potential of the mRNA loaded magnetic particles can be affected. The wetting behaviour of the buffer in the HGMS chamber plays also a role (see Section 3.1). Shaikh [50] measured particle size distributions of magnetic particles according to varied salt contents of sodium chloride and sodium dihydrogen phosphate in a range of 15.9–79 g·L^–1^. Less agglomeration occurred corresponding to higher salt concentrations. Paulus et al. [70] also stated that depending of the nature of salts and particles, agglomeration can be reduced. The PBS buffer has moderate salt content, while the lysis/binding buffer has high salt concentrations and contains an anionic detergent, sodium dodecyl sulfate (SDS). This substance has a stabilizing effect on the magnetic particle suspension, making magnetic separation with the lysis/binding buffer more difficult because agglomeration of the particles does not occur in the feed vessel. Therefore, the lysis/binding buffer is the most critical process step for magnetic separation design.

**FIGURE 7:**
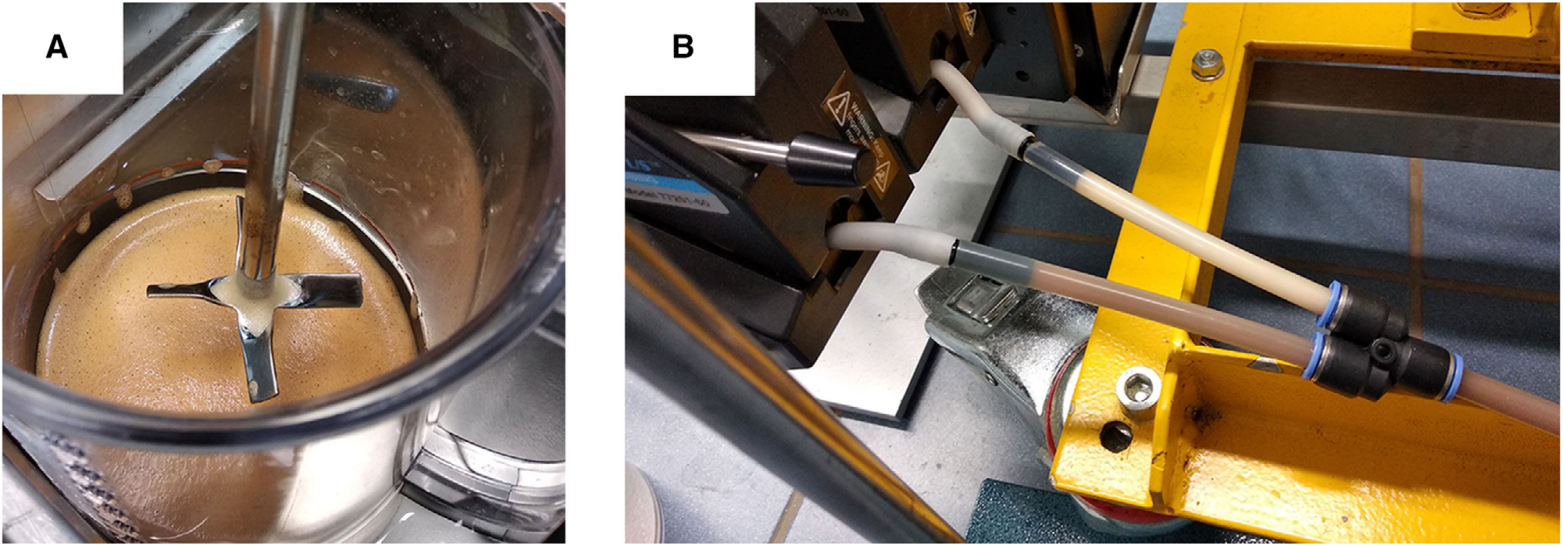
Foaming in the feed vessel (A) and accumulated foam (milky liquid in the upper tubing) while HGMS in lysis/binding buffer (B)

In addition, when detergents are used, increased foaming can be expected due to the lowered interfacial tension, which can also negatively affect magnetic separation. While stirring the suspension with lysis/binding buffer, there was foaming in the feed vessel at the interface of air and liquid (see Figure 7A). Also, air was emulsified within the bulk volume and partially suctioned by the pump. During HGMS, the air bubbles moved towards the separation chamber. Figure 7B shows an example of foam in the tubing during HGMS of magnetic beads in lysis/binding buffer. In case of foam being pumped through the separation chamber, already separated particles can be undesirably flushed out. This will result in decreased separation efficiency.

**FIGURE 8:**
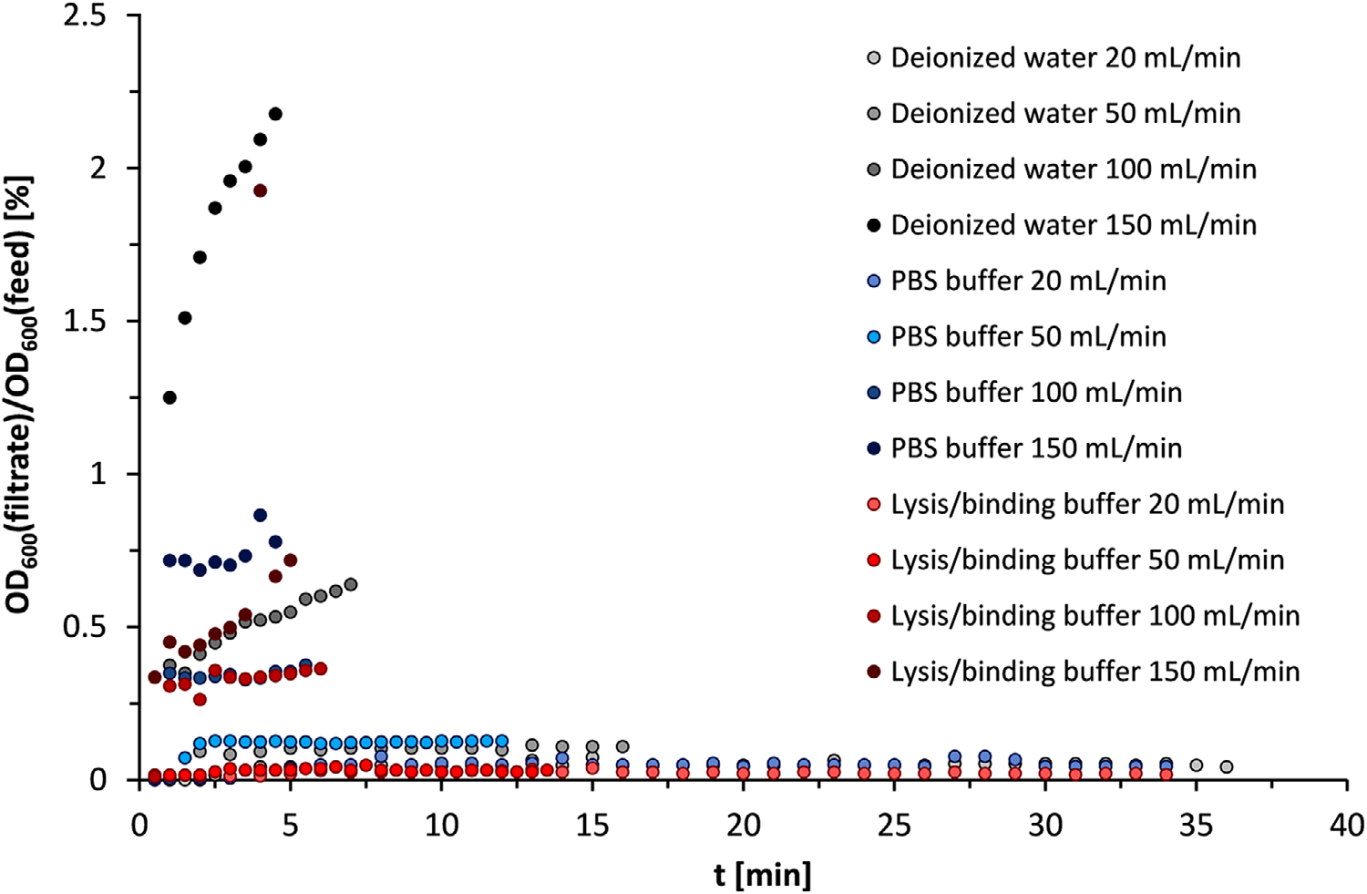
Separation of (m)RNA‐loaded oligo(dT)_25_ Dynabeads MyOne suspensions in binding/lysis buffer, PBS buffer, and deionized water with SU‐HGMS separation chamber at 20, 50, 100, and 150 mL·min^–1^

Based on the observations in Section 3.3.2, de‐agglomeration by ultrasonic was performed prior to each experiment as described in Section 2.2.4 to restore the original particle size distribution. The HGMS experiments with (m)RNA‐loaded oligo(dT)_25_ Dynabeads were then performed with volume flow rates of 20, 50, 100, and 150 mL·min^–1^. The results are shown in Figure 8. The particle mass retention capacity of the separation chamber could not be achieved with the available amount of magnetic beads of only 1.5 g. As expected, increasing the volumetric flow rate resulted in higher magnetic particle losses. In the case of lysis/binding buffer, an additional problem occurred in comparison with water and PBS buffer in foam or bubble formation during magnetic filtration due to the SDS content in lysis/binding buffer. As a result, magnetic particles that had already been deposited were partially discharged from the filter chamber, which led to a brief increase in the turbidity of the magnetic filtrate (see Figure 8, measuring point at t = 4 min for lysis/binding buffer 150 mL·min^–1^). At the same time, this possesses a measurement problem, since emulsified air bubbles increase the turbidity, making it difficult to quantify the magnetic particles.

In order to be able to make quantitative statements about the separation performance of the separation chamber, a separation efficiency η was calculated from the measured data. This is defined according to Equation 1 with the cumulative particle mass in the feed m_B,in_ and the cumulative particle loss in the magnetic filtrate m_B,out_. The particle mass in the feed and filtrate are calculated with the concentration of magnetic particles in the feed (c_B,0_) and filtrate (c_B_(t)), respectively, the volumetric flow rate $\dot{V}$ and the sampling intervals Δt.

(1) $$\eta =1-\frac{m_{B,\mathrm{out}}}{m_{B,\mathrm{in}}}=1-\frac{\sum c_{B}\left(t\right)\cdot \dot{V}\cdot \Delta t}{c_{B,0}\cdot \dot{V}\cdot t_{\mathrm{end}}}$$

Tables 1 and 2 show the cumulative particle losses based on the total mass of magnetically separated particles with the corresponding separation efficiencies. In addition, the recoveries are given in Tables 1 and 2, which show a closed mass balance. Even at 100 and 150 mL·min^–1^ particle retention can be considered sufficient, depending on the objective of the overall process. Moreover, very good separation efficiencies were observed at flow rates of 20 and 50 mL·min^–1^ in both lysis/binding buffer and PBS buffer. Remarkably, the cumulative particle losses in lysis/binding buffer were less than 0.01%, despite the de‐agglomerating and foaming effect of SDS. At volumetric flow rates up to 50 mL·min^–1^, the developed filter chamber is thus excellently suited for the separation of particles from the lysis/binding buffer during mRNA production.

**TABLE 1:** Mass balances of HGMS with agglomerated particles in dependency of volumetric flow rate in deionized water

Buffer	Volumetric flow rate [mL**·**min^–1^]	m_B,in_ [g]	m_B,out_ [g]	η [%]	Recovery [%]
Deionized water	20	1.224	<0.174 × 10^–3^	99.99	97.94
	50	1.383	0.658 × 10^–3^	99.95	85.51
	100	1.396	4.535 × 10^–3^	99.68	105.98
Average				99.87	96.48

**TABLE 2:** Mass balances of HGMS with non‐agglomerated particles in dependency of volumetric flow rate in lysis/binding buffer, PBS buffer, and deionized water

Buffer	Volumetric flow rate [mL**·**min^–1^]	m_B,in_ [g]	m_B,out_ [g]	η [%]	Recovery [%]
Lysis/binding	20	1.383	0.012 × 10^–3^	99.99	85.65
	50	1.106	0.015 × 10^–3^	99.99	95.37
	100	0.930	2.533 × 10^–3^	99.73	105.45
	150	1.241	7.622 × 10^–3^	99.39	83.19
Average				99.78	92.42
PBS	20	1.235	0.197 × 10^–3^	99.99	101.71
	50	1.077	0.890 × 10^–3^	99.92	95.07
	100	1.054	2.981 × 10^–3^	99.72	109.45
	150	1.315	8.253 × 10^–3^	99.37	102.47
Average				99.75	102.18
Deionized water	20	1.130	0.262 × 10^–3^	99.98	99.00
	50	1.284	0.932 × 10^–3^	99.93	96.87
	100	1.102	4.964 × 10^–3^	99.55	96.44
	150	1.077	17.188 × 10^–3^	98.40	106.58
Average				99.47	99.72

#### 3.3.4 Magnetic particle residues after cleaning the separation chamber with two‐phase flow

**FIGURE 9:**
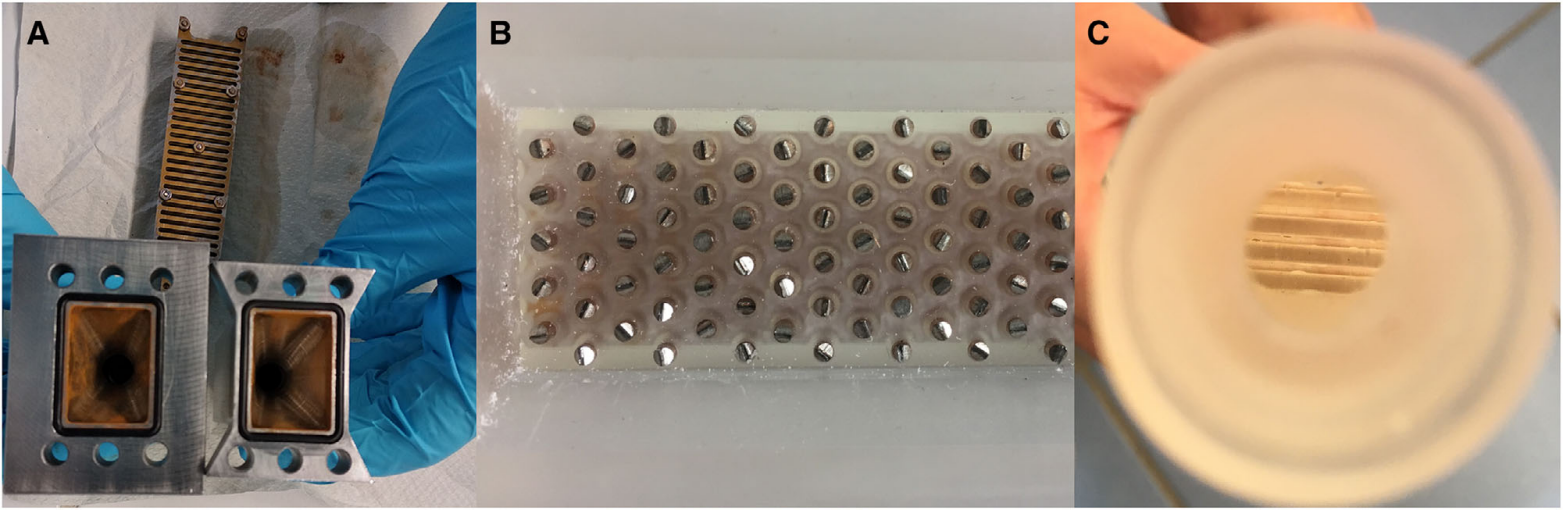
Residues of (m)RNA‐loaded oligo(dT)‐functionalized Dynabeads MyOne after cleaning in stainless steel separation chamber (A) and in 3D‐printed separation chamber in view from the side (B) and from above through the Tri‐Clamp connector (C)

After HGMS filtration, the magnetic particles retained inside the filter chamber were flushed out with a two‐phase flow of air in water as described in Section 2.2.1. Figure 9 shows a comparison of the cleaning results of the stainless steel filter matrix and the 3D‐printed filter chamber. When stainless steel is used, large particle deposits can be seen. These non‐rinsable residues not only represent particle losses, but would also lead to cross‐contamination of production batches. Therefore, a suitable cleaning‐in‐place would have to be performed additionally here. In the case of a SU‐HGMS filter chamber, cross‐contamination cannot occur due to the SU principle. Here, particle deposits in the separation chamber only represent particle losses. However, in contrast to the stainless steel, there were only a few local zones on the filter matrix where complete particle recovery did not occur. No pronounced depositions could be detected in the 3D‐printed separation chamber (see Figure 9B,C).

## 4 CONCLUDING REMARKS

With the developed SU‐HGMS separation chamber, mRNA manufacture can be realized at such a production scale, which should be sufficient for clinical trials. The standard operating procedures can be transferred directly from the corresponding laboratory protocols using magnetic particles. Increasing the amount of magnetic particles to be processed can be easily achieved by parallelization (up‐numbering). Using the build platforms of commercially available DLP 3D printers, the separation chamber including the liquid inlet and liquid outlet can be manufactured in one piece due to the developed design. All parts in contact with the product are made of the E‐Shell 600 material, for which the corresponding certification is already available (USP Class VI). Pre‐sterilization is also possible, so that such an SU‐HGMS separation chamber could be delivered to the user sterile. Because of the SU application, there is no need for extensive cleaning to avoid cross‐contaminations.

In addition to the very good separation efficiencies of >99%, which were achieved even with magnetic particles as small as the Dynabeads MyOne, the HGMS method is also convincing since it does not require solid/liquid separation as in preparative HPLC. The combination of these two aspects suggests that lower product losses can be expected in a process using the SU‐HGMS separation chamber than with the TFF/HPLC method. Of course, this statement still needs to be verified with future experiments that include cell debris and intracellular substances that affect viscosity. The realized volumetric flow rates can be compared to preparative HPLC systems with multi‐use columns as described in [11]. Larger volumetric flow rates can be realized if magnetic particles with a larger average particle size, such as the commercially available oligo(dT)‐functionalized 2.8 μm Dynabeads, are used. To further reduce product losses due to process time alone, throughput can also be increased by enlarging the magnetic field geometry, as this allows the cross‐section of the separation chamber to be increased.

**Nomenclature**
$\overset{}{V}$ [mL min^–1^]: Volumetric flow rate
c[g·L^–1^]: Concentration
d[m]: Diameter (of the stirrer)
d_3,50_[μm]: Median particle diameter of the volume distribution
m[g]: Mass
OD_600_[‐]: Turbidity at λ = 600 nm
t[s]: Time
T[°C]: Temperature
V[L]: Volume

**Greek symbols**
η[‐]: Separation efficiency
λ [nm]: Wavelength

**Indices**
0: Initial concentration of magnetic beads
B: Magnetic beads
in: Cumulative particle mass in the feed
out: Cumulative particle mass not retained in the separation chamber

## CONFLICT OF INTEREST

The authors have declared no conflicts of interest.

## Data Availability

The data that support the findings of this study are available from the corresponding author upon reasonable request.
